# Microstructure Evolution, Tensile/Nanoindentation Response, and Work-Hardening Behaviour of Prestrained and Subsequently Annealed LPBF 316L Stainless Steel

**DOI:** 10.3390/ma18051102

**Published:** 2025-02-28

**Authors:** Bohdan Efremenko, Yuliia Chabak, Ivan Petryshynets, Vasily Efremenko, Kaiming Wu, Sundas Arshad, František Kromka

**Affiliations:** 1 Physics Department, Pryazovskyi State Technical University, 49044 Dinpro, Ukraine; efremenko_b_v@pstu.edu (B.E.); chabak_y_g@pstu.edu (Y.C.); 2 Institute of Materials Research, Slovak Academy of Sciences, 04001 Kosice, Slovakia; ipetryshynets@saske.sk (I.P.); fkromka@saske.sk (F.K.); 3 International Research Institute for Steel Technology, Collaborative Innovation Center for Advanced Steels, Wuhan University of Science and Technology, Wuhan 430081, China; wukaiming@wust.edu.cn (K.W.); sundasarshad638@gmail.com (S.A.)

**Keywords:** 316L stainless steel, LPBF, cellular structure, prestraining, recrystallization annealing, mechanical properties, strain hardening rate

## Abstract

Additive manufacturing is increasingly used to produce metallic biomaterials, and post-processing is gaining increasing attention for improving the properties of as-built components. This study investigates the effect of work hardening followed by recrystallisation annealing on the tensile and nanoindentation behaviour of laser powder bed-fused (LPBF) 316L stainless steel, with the aim of optimising its mechanical properties. As-built and thermally stabilised (at 900 °C) specimens were prestrained in a uniaxially tensile manner at room temperature (0.12 plastic strain, ~75% of maximum work hardening) and subsequently annealed (at 900 °C or 1050 °C for 1 h). The microstructure and mechanical properties were then characterised by optical microscopy, SEM, EBSD, XRD, nanoindentation, and tensile testing. It was found that prestraining increased yield tensile strength (YTS) 1.2–1.7 times (to 690–699 MPa) and ultimate tensile strength (UTS) ~1.2 times (to 762–770 MPa), but decreased ductility 1.5 times. Annealing led to recovery and partial static recrystallisation, decreasing YTS (to 403–427 MPa), restoring ductility, and increasing the strain hardening rate; UTS and indentation hardness were less affected. Notably, the post-LPBF thermal stabilisation hindered recrystallisation and increased its onset temperature. Mechanical property changes under prestraining and annealing are discussed with respect to microstructure and crystalline features (microstrain, crystal size, dislocation density). All specimens exhibited ductile fractures with fine/ultra-fine dimples consistent with the as-built cellular structure. The combined treatment enhanced tensile strength whilst preserving sufficient ductility, achieving a strength–ductility product of 40.3 GPa·%. This offers a promising approach for tailoring LPBF 316L for engineering applications.

## 1. Introduction

316L-grade steel is a non-magnetic austenitic stainless steel that exhibits sufficient strength and high fracture/impact toughness combined with excellent corrosion resistance. The low carbon content in 316L (≤0.03 wt.%) makes it immune to sensitisation through carbide precipitation along the grain boundary, thus preventing intergranular corrosion [1]. Adding molybdenum to 316L significantly improves its resistance to pitting and crevice corrosion, especially in chloride-containing media, compared with other stainless steels and alloys [2,3]. This set of properties has caused the widespread use of 316L steel in various industries and production sectors, including medical and bioengineering [4]. 316L steel is produced through a conventional technological route consisting of casting and subsequent hot and cold rolling. However, in the last decade, the production of 316L products by additive manufacturing (AM) methods has become widespread [5,6,7,8].

316L steel is often used as a structural material, and so its strength is important for the reliable performance of components and structures. According to ASTM A276, the minimum values of the mechanical properties of conventionally manufactured 316L-grade steel are as follows: a yield tensile strength (YTS) of 182 MPa, an ultimate tensile strength (UTS) of 485 MPa, and a total elongation (TEL) of 35% [9]. The impact toughness of 316L steel reaches 350 J/cm^2^ at room temperature and up to 220 J/cm^2^ at –196 °C [10]. In compliance with standard ASTM F3184-16, additively manufactured 316L-grade steel should exhibit the following properties: YTS ≥ 215 MPa, UTS ≥ 485 MPa, and TEL ≥ 35% [11]. The above values of strength parameters (YTS, UTS) do not always comply with the operating conditions, limiting the applications of 316L. Therefore, improving the strength of 316L is a task that is gaining increasing relevance. This steel cannot be strengthened by thermal processing due to the wide range of gamma phase stability. However, solution heat treatment (HT) is feasible for 316L because it affects corrosion resistance [12,13,14] without affecting the anisotropic fatigue behaviour of AM specimens [15]. Typically, post-processing HT is used for 316L components to relieve the stresses remaining after the LPBF process [16]. During HT, the fine cellular/columnar microstructure of LPBF specimens evolves into an equiaxed austenitic structure, which causes a decrease in strength and deep stress relaxation [17]. As shown by Andreatta et al. [18], high-temperature HT does not affect the corrosion resistance of AM 316L steel, even after being maintained at 1100 °C for 8 h and 24 h.

Other (non-heat treatment) methods are used for strengthening 316L steel, mainly those associated with plastic deformation (bulk or surface). These are performed at different temperatures (from a cryogenic range to the conditions of “warm” deformation) [19,20,21,22]. Li et al. [23] applied 5% and 10% strain hardening at room temperature to wrought 316L steel and found that only yield strength responded significantly to prestraining, while the ultimate strength was almost unaffected. Multiaxial non-proportional cyclic “tension–torsion” prestraining of 316L steel resulted in a significant improvement in YTS with a reduction in TEL; moreover, the fatigue life under the lower strain amplitude of 0.2% was remarkably increased by 62% [20]. Kim et al. [19] found that a preliminarily formed heterogeneous structure comprising nano-sized σ-phase particles enhances the work hardening of 316L steel, enabling a tensile strength of 1 GPa. Even higher strengths can be achieved in 316L by prestraining at cryogenic temperatures [24,25] when deformation-induced martensite transformation (DIMT) is involved in steel hardening. The crucial importance of DIMT for advancing the mechanical and tribological properties of 316L, and other steels and alloys with an austenitic structure, is well acknowledged and reported in many works [26,27,28,29]. The activation of α′-martensite formation in 316L steel, under cryogenic thermo-mechanical processing, allowed an increase in its UTS, up to 1140 MPa, while maintaining a uniform elongation of 17% [24]. Prestraining (3.5%) at 4.2 K led to an increase in the YTS of about 45% due to the formation of multiple fine imperfections that served as sites for α′-martensite formation during tensile testing at room temperature [25]. The lattice defects (vacancies, dislocations) generated by deformation contribute to the trapping of hydrogen atoms in elastic fields [30].

With a growing interest in AM technologies for manufacturing 316L components, researchers’ efforts have focused on studying the effect of prestraining on the mechanical properties of AM-316L [31,32,33,34,35]. More often than not, the research objects are 316L specimens fabricated by laser powder bed fusion (LPBF) [36]. Sabzi et al. [32] identified the deformation mechanisms of LPBF 316L steel and highlighted the importance of twinning-induced plasticity in tensile behaviour. These findings were supported by Wang et al. [33], who reported the superior tensile behaviour of LPBF 316L specimens compared to their wrought counterparts, which was attributed to twinning and martensite transition, as the dominant deformation mechanisms of 3D-printed 316L steel. It was concluded that shear band formation and DIMT were responsible for a 20% increase in the hardness of LPBF 316L subjected to high-strain-rate deformation at room temperature [37]. The effect of Xe irradiation and hydrogen embrittlement on the strain hardening mechanism of LPBF stainless steels (304L and 316L) was analysed in [38,39].

The plastic deformation mechanism and strain hardening rate of AM-316L can be attributed to AM process parameters [40], crystallographic texture [41], and cellular and hierarchical structures [33]. As shown in [42], the printing scanning strategy affects the mechanism of compressive plastic deformation in AM-316L: under build angles of 90° and 67.5°, twinning-induced plasticity occurs. Under 0°-scanning, strong crystallographic textures occur, causing anisotropic deformation. The peculiarities of the solidification of the LPBF 316L microstructure strongly influence recrystallisation kinetics [43,44]. It was concluded [44] that recrystallisation kinetics depend on the amount of low-angle grain boundaries (LAGBs) formed during the LPBF process: high LAGB density promotes recrystallisation due to an increase in stored energy near grain boundaries. The approach of tailoring recrystallisation and, thus, the microstructure of 3D-printed 316L via a laser scanning strategy, was proposed in [45].

Surface straining allows the creation in 316L of gradient (surface → bulk) structures such as “ultra-fine nanocrystals → ultra-fine grain → coarse sub-grain”. The most commonly used techniques for the surface strengthening of 316L are severe shot peening (SSP), ultrasonic shot peening (USP), ultrasonic nanocrystal surface modification (UNSM), and laser shock peening (LSP) [46,47,48]. LSP increases the surface hardness of LPBF 316L steel to 4.87 GPa, providing a 41.4% increase in wear resistance [47] and a corresponding enhancement of tensile strength [48]. UNSM improves the mechanical properties and fatigue life of AM 316L steel, especially when UNSM undergoes stress-release heat treatment [49]. The positive effect of UNSM is attributed to extreme grain refinement induced by shear strain and, also, the reduction in micro-cracks resulting from surface machining [50]. Kim et al. [49] proposed a new UNSM-based strategy to reduce defects intrinsic to LPBF-fabricated 316L steel through the elimination of subsurface pores and surface roughness control. The feasibility of using UNSM to increase the sliding wear resistance and corrosion resistance of LPBF 316L steel was shown by Amanov in [51].

Despite the above data, there is a lack of work devoted to advancing AM 316L steel through its work-hardening and subsequent recrystallising heat treatment. To fill this gap, this paper comprehensively investigates the possibility of controlling the microstructure and mechanical properties of LPBF 316L steel by combining dislocation engineering (prestraining) and grain refinement during static recrystallisation. Additional attention is paid to studying the effect of the initial (before prestraining) structure of LPBF specimens (which are “cellular” in the as-built state or “polyhedral” after thermal stabilisation) on the kinetics of recrystallisation of cold-worked austenite grains.

## 2. Materials and Methods

**Figure 1:**
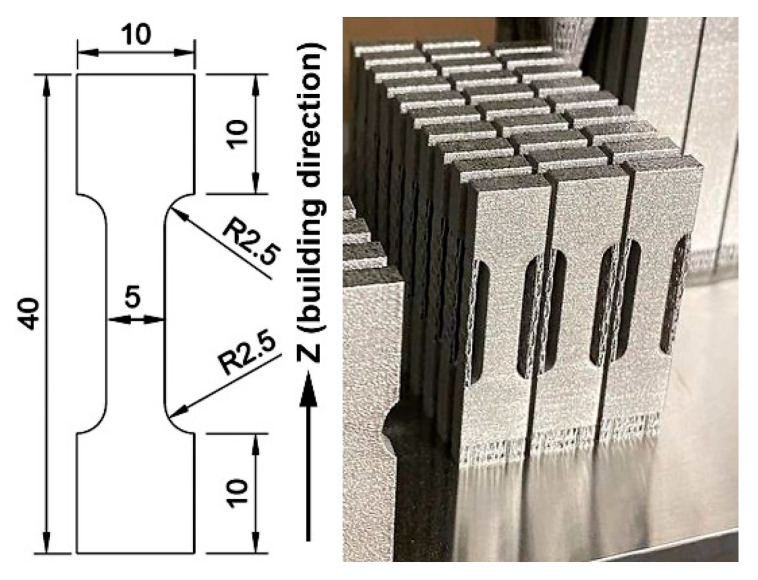
The shape, sizes, and view of the LPBF-fabricated specimens of 316L steel (thickness of the specimens is 4 mm).

Flat 4 mm thick “dog-bone”-shaped specimens of 316L steel were additively manufactured by the LPBF process using an “Alfa-150D” 3D-printer (Additive Laser Technology Ukraine, Dnipro, Ukraine) while applying the following parameters: a laser power of 350 W, a laser spot size of 100 µm, a printing speed of 950 mm/s, 200 µm hatch spacing, 80 µm layer thickness, and a rotation angle of 67°. The specimens were printed in a vertical position (according to ISO/ASTM 52900 [52]), i.e., with the Z direction aligned with the specimen’s long axis. The shape, sizes, and views of the as-built specimens are shown in Figure 1. Gas-atomised powder, with a particle size of 15–45 µm, was used as the powder feedstock for the LPBF process. The chemical composition of the LPBF-printed specimens was determined using the spark optical emission spectrometer “Labspark 1000” (NCS Testing Technology Co., Ltd., Beijing, China). It was found to be in full compliance with ASTM F3184-16 (Table 1).

**Figure 2:**
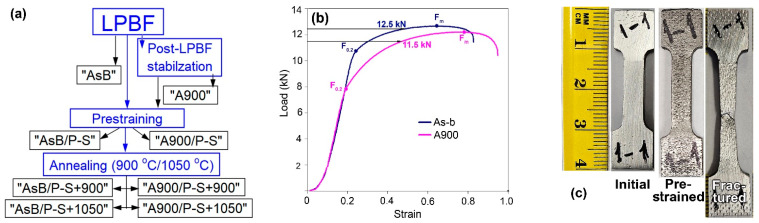
(**a**) The scheme of the processing sequence and the designation of the specimens; (**b**) tensile curves for the “AsB” and “A900 specimens; (**c**) views of the specimens in the initial state and after prestraining and fracturing under tensile testing.

Two groups of the LPBF 316L specimens were studied: (a) specimens in an as-built state (denoted as “AsB”); and (b) post-LPBF specimens, thermally stabilised by being held at 900 °C (denoted as “A900”). The scheme of the processing sequence and the designation of the specimens is depicted in Figure 2a. The duration of the stabilising holding was selected to be 5 h to ensure the full elimination of the as-built cellular structure, which is a diffusion-driven process [53]. The research approach included preliminary plastic prestraining (P-S) at room temperature followed by annealing at 900 °C or 1050 °C to stimulate the structure’s evolution through recovery and recrystallisation. Before prestraining, the offset yield load (F_0.2_) and ultimate tensile load (F_m_) values were found for the specimens in their initial state. These were as follows: F_0.2_ =10.89 kN and F_m_ =13.04 kN (for the “AsB” specimens); F_0.2_ = 7.65 kN and F_m_ =12.70 kN (for the “A900” specimens). Prestraining was performed by uniaxial tensioning to the total strain of ~0.3, reaching a load corresponding to 75% of the load increase from F_0.2_ and F_m_ (as shown in Figure 2b); the maximum prestraining loads were 12.5 kN and 11.5 kN for the “AsB” and “A900” specimens, respectively. The residual strain was ~0.12. Images of the specimen before and after prestraining are presented in Figure 2c. Then, the prestrained specimens were annealed at 900 °C or 1050 °C for 1 h and cooled in still air. The designations of the specimens were as follows: (a) prestrained (“AsB/P-S” and “A900/P-S”); (b) prestrained and annealed (“AsB/P-S+900(1050)” and “A900/P-S+900(1050)”) (Figure 2a).

Heat treatments (both post-LPBF stabilising and recrystallisation annealing) were carried out in an electric muffle furnace with a protective atmosphere of technical-grade argon. Uniaxial prestraining and tensile testing (to fracture) were performed at room temperature using an electro-mechanical “TiraTest 2300” machine (TIRA GmbH, Schalkau, Germany) at an engineering strain rate of 1.7·10^−3^ s^−1^ (selected to ensure slow loading to prevent the early failure of 3D-printed specimens). Before the testing, the as-built roughness was removed from the specimen’s surface by sandpaper grinding to R_a_ = 0.2 µm. Nanoindentation was carried out using a “Nano Indenter G200” device (Agilent Technologies, Santa Clara, CA, USA) device. The Bercovich-type diamond pyramid was indented into the surface with a displacement speed of 10 nm/s to reach a maximum load of 500 mN; a grid of 10 × 10 with a 50 µm step was used to make one hundred imprints on each specimen, with the subsequent averaging of the results. Microhardness was measured by the Vickers method using a “LM700AT” hardness tester (LECO, St. Joseph, MI, USA) at a load of 25 g. The low load was chosen to take into account the need to measure hardness in the area in close proximity to the rapture zone of tensile specimens.

Specimens for the microstructure observation were prepared according to the routine metallographic procedure of grinding with emery SiC papers of a decreasing abrasive size and finishing with Al_2_O_3_ aqueous suspensions. The mirror-polished specimens were etched with “Aqua Regia” solution (a 1:3 mixture of HNO_3_ and HCl, respectively) for up to 1 min. An “GX71” optical microscope (OM) (Olympus, Tokyo, Japan) and a “JSM-7000F” electronic scanning microscope (SEM) (JEOL, Tokyo, Japan) were used for microstructure characterisation and fracture surface observation. The area fraction of the recrystallised structure was calculated using ten randomly selected OM images showing an area of 325 µm × 425 µm, with the subsequent averaging of the results. Electron backscatter diffraction (EBSD) analysis was performed using the “Symmetry S3” System (Oxford Instruments, High Wycombe, UK), combined with an “Apreo S Hivac” field emission SEM (Thermo Fisher Scientific, Waltham, MA, USA). The EBSD signal was obtained at 20 kV with a 1 µm step size. X-ray diffraction (XRD) was performed using an “X’Pert PRO” diffractometer (PANalytical, Malvern, UK) to study the crystalline status of the specimens. XRD measurements were carried out under the following parameters: Cu-Kα radiation, a voltage of 40 kV, a tube current of 50 mA, a scan step of 0.033°, and a scan speed of 0.069°·s^−1^.

## 3. Results

### 3.1. Mechanical Behaviours Assessment

#### 3.1.1. Tensile Testing

**Figure 3:**
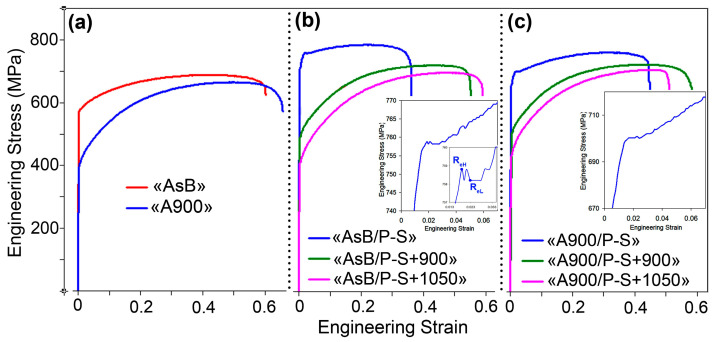
Engineering “stress–strain” curves for the specimens: (**a**) in the initial state (before prestraining); (**b**) the group “AsB”; (**c**) the group “A900”.

**Figure 4:**
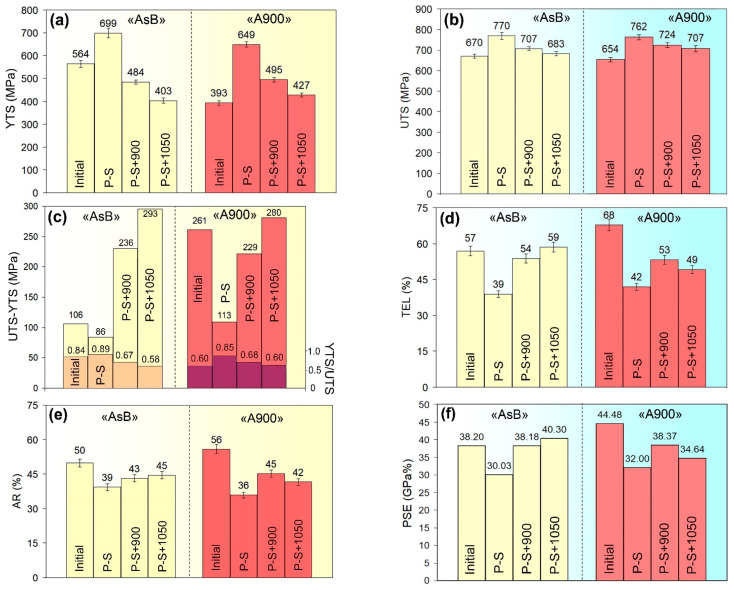
Mechanical properties of the specimens depending on processing mode: (**a**) YTS; (**b**) UTS; (**c**) difference (UTS–YTS) and yield strength ratio (YTS/UTS); (**d**) TEL; (**e**) AR; (**f**) PSE (designations: initial—before prestaining; P-S—prestraining; P-S+900 °C and P-S+1050 °C—prestraining and subsequent annealing at 900 °C or 1050 °C, respectively).

Figure 3 shows the engineering “stress–strain” curves for the LPBF 316L steel when processed by different schemes. Figure 3a refers to the specimen in the initial (before prestraining) state. It can be seen that, in the as-built specimen, plastic deformation began at a significantly higher load than that in the thermally stabilised specimen, while the latter displayed greater total elongation. Meanwhile, both specimens demonstrated similar values of ultimate load with a slight advantage for the “AsB” specimen. Accordingly, the as-built specimen showed much higher YTS compared with the thermally stabilised specimen (564 ± 15 MPa vs. 393 ± 10 MPa, respectively, Figure 4a) and rather similar UTS values (670 ± 10 MPa and 654 ± 11 MPa, respectively, Figure 4b). The thermally stabilised specimen had a significant advantage in ductility, displaying a TEL of 68 ± 3% and an area reduction (AR) of 56 ± 2%, which are 11 points and 6 points better, respectively, than those of the as-built specimen (Figure 4c,d). These values of mechanical properties significantly exceed the minimum values stipulated by ASTM F3184-15 for the LPBF-manufactured 316L steel.

The curves of the prestrained specimens of both groups demonstrated a higher offset of deformation load and a lower elongation to rupture compared to the initial state (Figure 3b,c). This behaviour indicated a significant increase in the strength of both groups of specimens, with a trade-off decrease in their plasticity. Notably, the prestrained specimens exhibited a yield plateau (shown in Figure 3b,c), while the curve of the “AsB” specimen featured upper and lower yield points and the “pop-in” phenomenon on the ascending part of the curve (this refers to stress fluctuations at a constant strain due to the generation and motion of multiple dislocations to accommodate the applied strain [54]). Due to prestraining, YTS increased relative to the initial state, rising by 135 MPa (1.24 times) for “AsB” specimens and by 256 MPa (1.65 times) for “A900” specimens, reaching 699 ± 19 MPa and 649 ± 12 MPa, respectively. The tensile strength also increased, but to a lesser extent: the UTS values were almost the same (~770 MPa) for both groups of specimens. Strain hardening caused ductility deterioration: TEL and AR decreased by about 1.5 times compared to the initial state in both groups of specimens (Figure 4d,e).

Post-prestraining annealing resulted in a change in the shape of the “engineering stress–strain” curves: they lost the yield strength plateau and did not exhibit the “pop-in” behaviour. This was accompanied by decreasing yield loads and ultimate load values and increasing elongation to fracture compared with the prestrained specimens. These tendencies increased with the annealing temperature. Accordingly, a decrease in the strength indices occurred, while yield strength declined more significantly. In the “AsB” group of specimens, the YTS dropped below the as-built level (by 80 MPa and 161 MPa after annealing at 900 °C and 1050 °C, respectively). On the contrary, in the “A900” group of specimens, work hardening and annealing provided an increase in the yield strength relative to the non-prestrained state. The ultimate strength was slightly affected by annealing: the UTS values maximally decreased to 683 ± 10 MPa (“AsB” specimens) and 707 ± 15 MPa (“A900” specimens). In contrast to strength, the ductility of the prestrained specimens was improved by annealing, and to a greater extent—in the specimens of the “AsB” group, TEL increased from 39 ± 2% (P-S) to 59 ± 2% (P-S+1050 °C), hence exceeding the level of the as-built state. In the specimens of the “A900” group, TEL only increased by 7–11 points (from 42 ± 1% to 49–53%). Similarly, with the increase in the annealing temperature, area reduction increased, but not as notably as TEL, and so the initial level was not reached here. Figure 4d,e show that the ductility of the specimens of the “AsB” group responded to annealing much better. For the specimens of the “A900” group, the initial ductility was not recovered by post-prestraining annealing.

Figure 4c demonstrates the capacity of the steel to harden under plastic deformation depending on the processing mode. Here, two parameters were used that were derived from tensile testing: the difference between UTS and YTS, and the “YTS/UTS” ratio. These parameters change in an inversely proportional manner to each other; they are used as direct and indirect indicators of the steel’s work-hardening capacity, respectively. Before prestraining, the “AsB” specimens demonstrated a lower work-hardening rate: the difference (UTS–YTS) and YTS/UTS were 106 MPa and 0.84, respectively. In contrast, the “A900” specimens displayed a higher propensity for work-hardening (up to 2.5 times): (UTS–YTS) = 261 MPa and YTS/UTS = 0.60. After prestraining, the hardening behaviour deteriorated in both groups of specimens, most sharply in the “A900” group, where the difference (UTS–YTS) decreased to 86 MPa and 113 MPa for the “AsBs” and “A900s” groups, respectively. Post-prestraining annealing caused a sharp increase in (UTS–YTS) in both groups of specimens, resulting from a decrease in YTS and an increase in TEL. Notably, in the annealed state, both groups of specimens were nearly equal, with respect to the difference (UTS–YTS) and the “YTS/UTS” ratio, with a slight advantage for the “AsBs.”

#### 3.1.2. Nanoindentation

**Figure 5:**
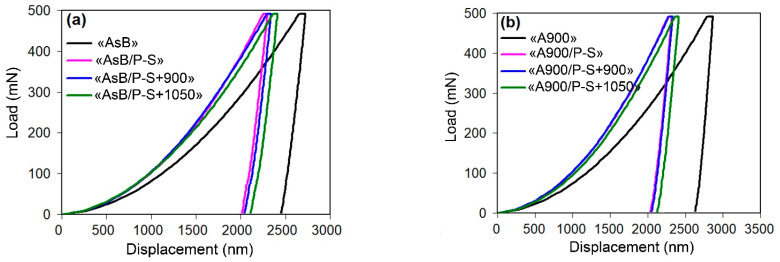
The representative “load–displacement” curves derived from the nanoindentation test: (**a**) the specimens of an “AsB” group; (**b**) the specimens of an “A900” group.

The micromechanical properties of the steel were evaluated using nanoindentation testing. The representative “load–displacement” curves, corresponding to the mean hardness value for each processing mode, are depicted in Figure 5. It can clearly be seen that the curves referring to the initial state of the specimens are distinguished by greater penetration of the indenter into the surface, indicating lower resistance to plastic deformation compared to other specimens that undergo prestraining.

**Figure 6:**
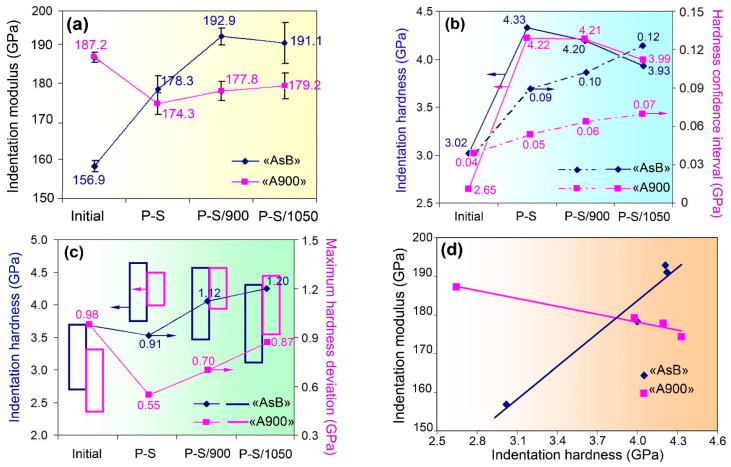
The results of the nanoindentation test. The effect of the processing mode on the evolution of (**a**) the indentation modulus; (**b**) the average value of indentation hardness and the hardness confidence interval; (**c**) the scatter of the hardness values (shown by the rectangles) and maximum hardness deviation; (**d**) “indentation hardness–indentation modulus” correlation.

The results derived from the nanoindentation testing (the values of indentation modulus and indentation hardness) are depicted in Figure 6. The average indentation modulus of the as-built specimen was rather low—156.9 ± 1.4 GPa (Figure 6a)—but, after prestraining, it increased to 178.3 ± 3.7 GPa, and it further stabilised at 191–193 GPa after post-prestraining annealing. The specimen of the group “A900” showed an average modulus of 187.2 ± 1.1 GPa in the initial state; after prestraining and subsequent annealing, it slightly decreased and stabilised at 174–179 GPa.

The evolution of indentation hardness is presented in Figure 6b. In the initial state, the “AsB” specimen was harder compared with the “A900” specimen (3.02 ± 0.02 GPa and 2.65 ± 0.03 GPa, respectively). Subsequently, both groups of specimens showed similar hardness profiles depending on the processing mode: the average hardness sharply increased to 4.2–4.3 GPa after prestraining followed by a gradual decrease to 3.9–4.0 GPa as the annealing temperature increased (Figure 6b). However, a more detailed analysis revealed differences in the evolution of the hardness of both groups of specimens. As can be seen from Figure 6b, the transition from the initial state to annealing at 1050 °C was accompanied by an increase in the hardness confidence interval, which increased more intensively in the specimens of the “AsB” group, leading to an almost twofold difference between “AsBs” and “A900s” (0.07 and 0.12, respectively). Figure 6c illustrates the change in the scatter of hardness values depending on the processing mode. It can be concluded that, starting from the initial (non-prestrained) state, the “AsBs” are characterised by a larger scatter of hardness values and a lower minimum value within the scatter interval. This reflects greater structural heterogeneity in the “AsB” specimens formed under different processing compared to the “A900” specimens.

Figure 6d shows that indentation hardness and indentation modulus display a close correlation, which can be described by linear regression equations. However, the dependence of hardness on the modulus differs significantly for the “AsB” and “A900” groups of specimens. For the “AsB” specimens, this dependence is positive (ascending), while for the “A900” specimens, a decrease in modulus is observed as hardness increases. Accordingly, the aforementioned relationship is represented by different equations for the “AsB” (1) and “A900” (2) specimens, which are as follows:

*y* = 28.27*x* + 70.62   R^2^ = 0.95, (1)

*y* = –6.86*x* + 205.68   R^2^ = 0.95. (2)

where *x* and *y* represent indentation hardness (GPa) and indentation modulus (GPa), respectively.

The high coefficient of determination (R^2^ = 0.95) suggests that (1) and (2) accurately predict the relationship between the variables. Equations (1) and (2) differ greatly in the coefficient of *x*, which reflects the slope of the curve, i.e., the response of the modulus to changes in hardness. As can be seen, for the “AsB” specimens, this response is four times greater than that for the “A900” specimens. At hardness values of 3.9–4.3 GPa, the modulus of the “AsB” specimens is, on average, 14 GPa higher than that of the “A900” specimens, suggesting that this behaviour is attributable to the cellular structure of the “AsB” specimens. Cell boundaries are dislocation walls [55] that hinder dislocation glide and lead to rapid pile-up under plastic strain. As a result, the structure acquires not only increased hardness (resistance to plastic deformation) but also a larger modulus (resistance to elastic strain). This finding corresponds to the conclusion of Guo et al. [56] that the cellular structure of 3D-printed 316L exhibits a large capacity for strain hardening.

### 3.2. Microstructure Observation

**Figure 7:**
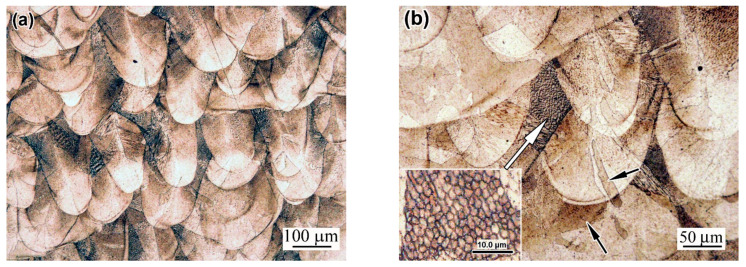

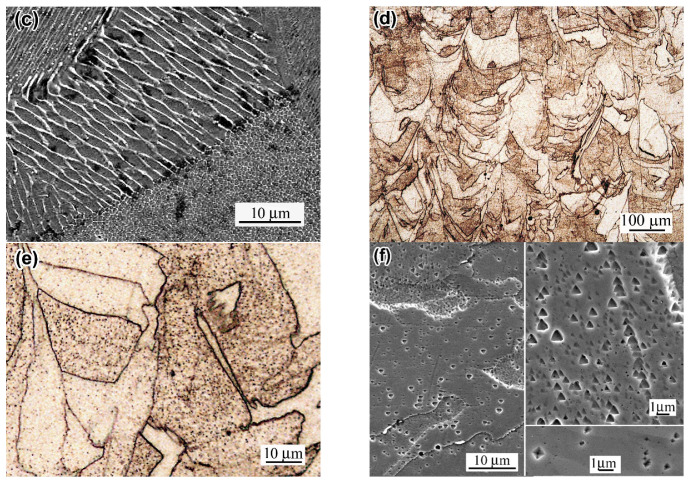
The microstructure of the LPBF 316L steel in the initial state (before prestraining): (**a**–**c**) as-build; (**d**,**e**) thermally post-stabilised at 900 °C; (**f**) etching pits ((**a**,**b**,**d**,**f**)—OM; (**c**,**e**)—SEM).

Figure 7 depicts the initial microstructure of the LPBF 316L steel. The as-built structure exhibits characteristic features of the LPBF, such as a “fish-scale” pattern composed of melt pools retained after the selective melting of the powders under the laser beam (Figure 7a). The melt pools are aligned in rows positioned perpendicular to the building direction. They have a semi-spherical, elongated shape with a width of up to 150 µm and a depth of up to 200 µm (Figure 7b). Furthermore, the structure exhibits intrinsic SLM-originating defects such as “lack-of-fusion” pores (with an area fraction of 1.5–2.0%) and non-metallic inclusions. Each melt pool exhibits a fine columnar/cellular structure, characteristic of LPBF-fabricated alloys (repeatedly described in previous works [5,36,43]). This consists of differently oriented bundles of fine, elongated crystals over tens of micrometres long (Figure 7c). The cross-sectional sizes of the columnar crystals varied from about 0.2 µm to 1.5 µm. Fine cells were revealed by SEM observation, while coarser ones were even seen in OM, in the junction areas between the melt pools, as shown by white arrow in the insert in Figure 7b. The grain boundary pattern was discerned and was superimposed on the melt pools, where epitaxial grains were noted to extend through several neighbouring melt pools (shown by black arrows in Figure 7b).

After annealing at 900 °C, the “as-built” pattern of the structure was generally retained, and the contours and boundaries of the former melt pools were clearly discernible (Figure 7d). However, significant changes occurred in the structure, which lost its columnar/cellular character. Instead of columnar crystal/cell colonies, vast grain areas formed with a relatively smooth surface (Figure 7e). These areas featured numerous etch pits of triangular or tetrahedral shapes covering the surface of the grains (Figure 7f). The pits were unevenly distributed in different grains; in some places, they lined up in rows, both within the grains and along their boundaries (as in the upper right-hand side of Figure 7f). Due to the presence of multiple pits, the grains acquired a mottled pattern which was even seen using the optical microscope (Figure 7e).

**Figure 8:**
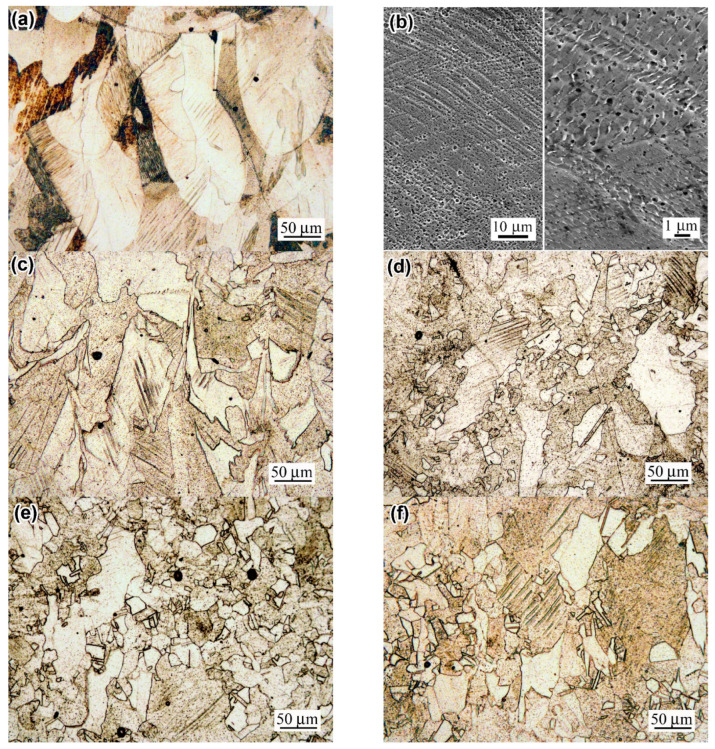
The evolution of the microstructure of the specimens of an “AsB” group: (**a**,**b**) “AsB/P-S”; (**c**,**d**) “AsB/P-S+900”; (**d**–**f**) “AsB/P-S+1050” ((**a**,**c**–**f**)—OM, (**b**)—SEM).

**Figure 9:**
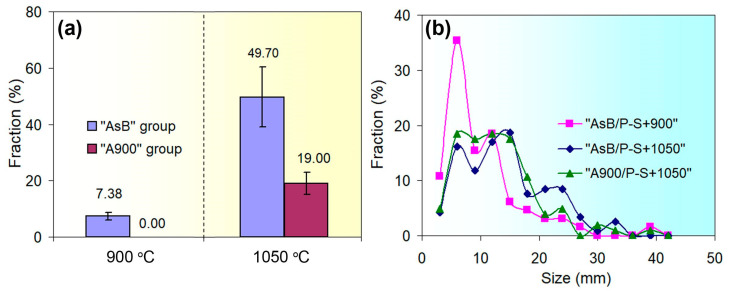
(**a**) The average area fraction of recrystallised grains; (**b**) the recrystallised grain size distribution depending on the annealing temperature.

The structure of a prestrained “AsB” specimen is illustrated in Figure 8a,b. Since the melt pools were initially oriented along the printing direction (i.e., the longitudinal axis of the specimen), upon prestraining, they were found to stretch up to 250 µm long with a simultaneous decrease in thickness to approximately 100 µm (Figure 8a). Slip bands (up to 1.5 µm wide) appeared inside certain melt pools, spreading into adjacent melt pools, and etch pits appeared on the surface between them (Figure 8b). The melt pools themselves retained their cellular structure, although within individual colonies of columnar crystals, mutual displacement (shift) occurred, which is caused by the appearance of slip bands. After post-prestraining annealing at 900 °C, the structure of the “AsB” specimen became similar to the initial structure of the “A900” specimen (i.e., similar to that of post-LPBF thermal stabilising). It lost the cellular pattern, but maintained the general “melt pools” pattern and presented multiple etch pits (Figure 8c). In this case, the structure became differentiated, being divided into non-recrystallised areas (Figure 8c) and partially recrystallized areas (Figure 8d). In the latter, the emergence of fine equiaxed grains with annealing twins was recorded (they were almost free of etching pits). The average area fraction of recrystallised grains in the “AsB/P-S+900” specimen was estimated as 7.38% (Figure 9a), while their size varied in the range of 1–40 µm, with an average value of 8.8 ± 1.5 µm; among them, grains 3–12 µm in diameter prevailed (Figure 9b).

When the temperature of post-prestraining annealing was 1050 °C, recrystallisation developed to a greater extent: recrystallised grains were recorded over the entire area, and their average area fraction increased significantly—up to 49.7%. Examples are given in the images shown in Figure 8e,f (the recrystallised grains amounted to 69% and 26%, respectively). Moreover, they grew, and their average size increased almost twofold (up to 14.58 ± 1.4 µm). It is noteworthy that despite the development of static recrystallization at 900 °C and 1050 °C, the slip bands that appeared under the prestraining stage were partially retained, demonstrating the stability of the work-hardened structure.

**Figure 10:**
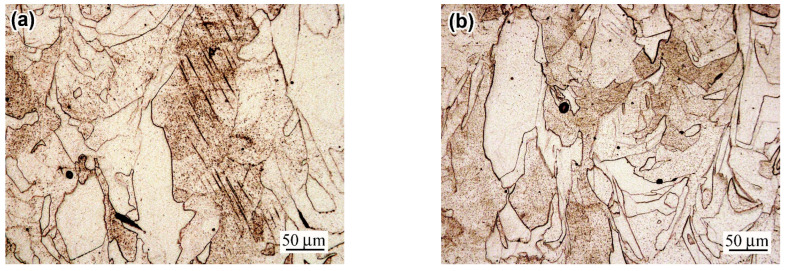

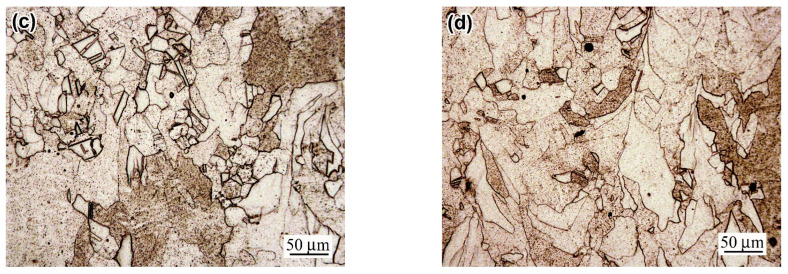
The evolution of the microstructure of the specimens of an “A900” group: (**a**) “A900/P-S”; (**b**) “AsB/P-S+900”; (**c**,**d**) “A900/P-S+1050” (OM).

In the specimens of the “A900” series, prestraining caused the deformation of grains and the appearance of slip bands in some of them (Figure 10). After post-prestraining annealing at 900 °C, no recrystallised grains were revealed in the structure, unlike the “AsB” specimen (Figure 10b). The static recrystallisation only started in the “A900s” at 1050 °C (Figure 10c,d), leading to the formation of new equiaxed grains of 2–42 µm in diameter (13.6 ± 1.4 µm, on average) (Figure 9b). Figure 10c,d illustrate that one of the areas is twice that of the other in terms of the recrystallised grains area fractions (measured as 29% and 14%, respectively). The average area fraction of the 1050 °C recrystallised structure was 19% (Figure 9a).

### 3.3. XRD Analysis

**Figure 11:**
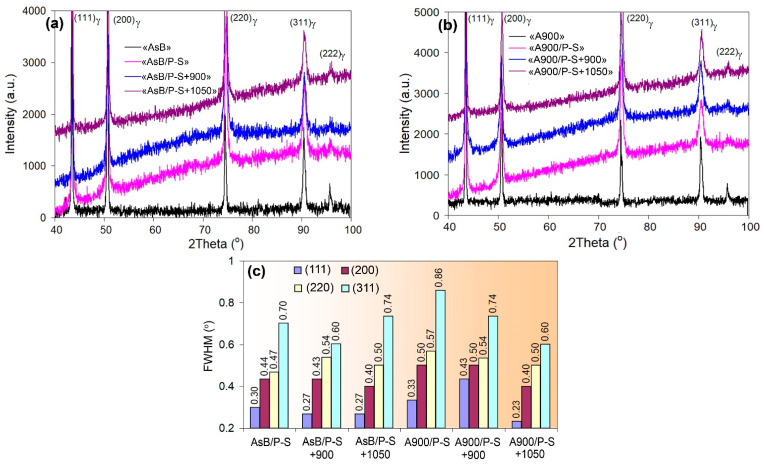
XRD patterns of the specimens of the: (**a**) “AsB” group and (**b**) “A900” group. (**c**) Full width at half maximum (FWHM) of the diffraction peaks.

Figure 11a,b illustrate the X-ray diffraction patterns of the specimens of the “AsB” and “A900” groups, respectively. All the XRD patterns only featured the peaks of the FCC phase, which was a γ-solution of Cr, Ni, and Mo in iron (austenite). XRD patterns were used to estimate the microstructural evolution by analysing the full width at half maximum (FWHM) of the diffraction peak, which reflected the crystalline/strain state of the metal [57]. The measured FWHM values of the diffraction peaks (111)γ, (200)γ, (220)γ, and (311)γ are presented in Figure 11c. Within each group of specimens, the lowest and highest FWHM values (for each specific peak) were attributed to the initial and prestrained states, respectively. In a prestrained state, the broadening of the peaks was higher in the specimens of the “A900” group.

### 3.4. EBSD Characterisation

**Figure 12:**
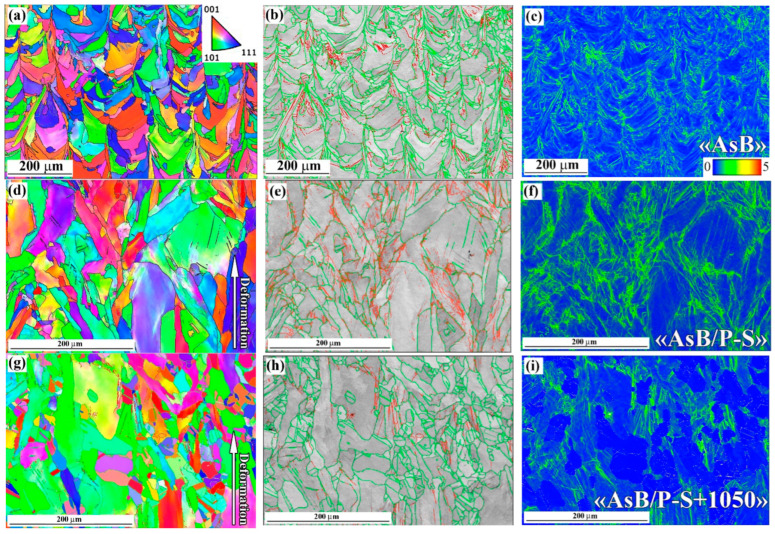
The results of the EBSD analysis of the specimens “AsB” (**a**–**c**), “AsB/P-S” (**d**–**f**) and “AsB/P-S+1050 °C” (**g**–**i**): ((**a**,**d**,**g**) are IPF maps; (**b**,**e**,**h**) are the (BC + GB) maps (green lines are the HAGB, red lines are LAGB); (**c**,**f**,**i**) are KAM maps).

**Figure 13:**
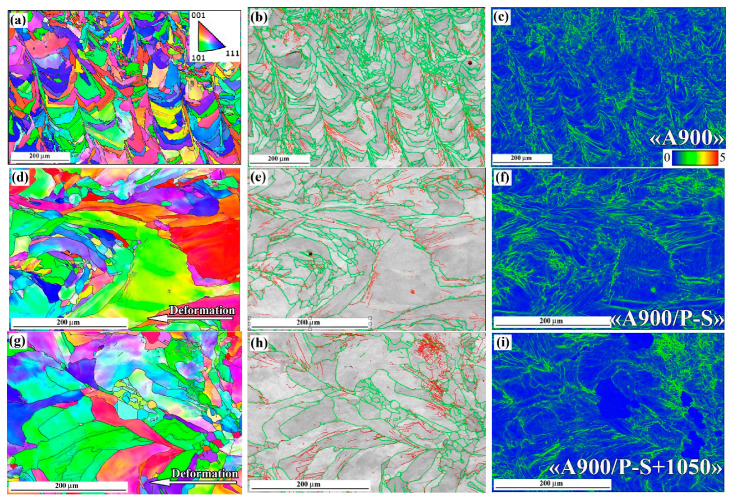
The results of the EBSD analysis of the specimens “A900” (**a**–**c**); “A900/P-S” (**d**–**f**) and “A900/P-S+1050 °C” (**g**–**i**): ((**a**,**d**,**g**) are IPF maps; (**b**,**e**,**h**) are the (BC + GB) maps (green lines are the HAGB, red lines are LAGB); (**c**,**f**,**i**) are KAM maps).

**Figure 14:**
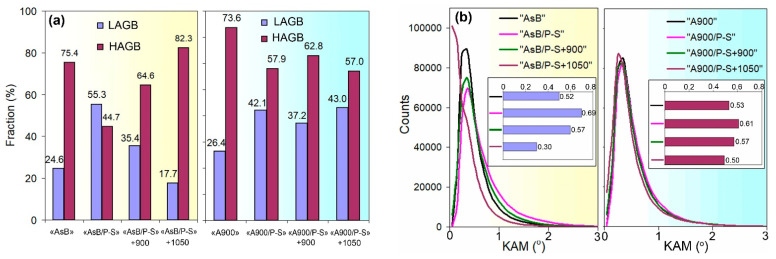

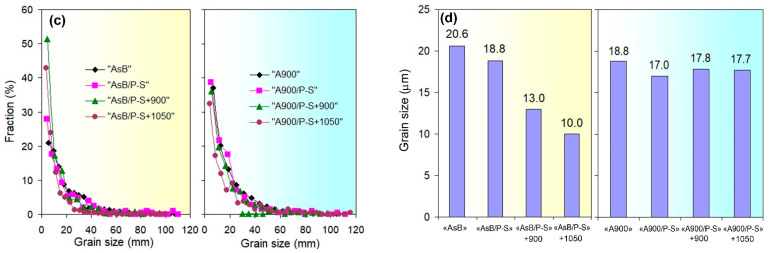
EBSD data of grain status and kernel misorientation: (**a**) LAGB and HAGB distributions; (**b**) KAM distributions; (**c**) density distributions of grain size (equivalent circle diameter); (**d**) the average grain size values. The average KAM values are presented in the inserts in Figure (**b**).

The results of the EBSD characterisation of the specimens are presented in Figure 12 and Figure 13 and summarised in Figure 14. The inverse pole figure (IPF) colour map of the “AsB” specimen (Figure 12a) shows that the grain’s arched shape directly follows the melt pool pattern. The IPF revealed no preferential crystallographic texture in the “AsB” specimen; instead, a significant spatial misorientation of the neighbouring grains was observed. As follows from the grain boundary (GB) map (Figure 12b), the grains were mostly divided by high-angle (>10°) grain boundaries (HAGBs), the fraction of which reached 75.4% (Figure 14a). Low-angle grain boundaries (LAGBs) were mainly spread within the grains. The average kernel misorientation (KAM) map is presented in Figure 12c; the KAM value in the “AsB” specimen varied from 0° to 4.95°, with a mean value of 0.52° (Figure 14b). The prestraining of the “AsB” resulted in the grains extending without acquiring any notable texture (Figure 12d). The HAGB/LAGB ratio changed in favour of the latter due to the appearance of new LAGBs inside the grains, mainly along the slip bands (Figure 12e). Accordingly, the average KAM value increased to 0.69, exhibiting a non-uniform distribution of local misorientation, localised mainly along the LAGBs (Figure 14b). The post-prestraining annealing of the “AsB” specimen caused the appearance of small recrystallised grains (Figure 12g) separated from the parent matrix by HAGBs (Figure 12h); LAGBs remained present in a lower amount, being mostly associated with the slip bands. Accordingly, after annealing at 900 °C and 1050 °C, the proportion of HAGBs sharply increased to 64.6% and 82.3%, respectively, while the average KAM value remarkably decreased to 0.57° and 0.32°, respectively (Figure 14b).

The EBSD response of the non-prestrained “A900” specimen was similar to that of the “AsB” specimen, presenting differently oriented “melt pool”-shaped grains (Figure 13a), with a majority of HAGBs (73.6%, a HAGBs:LAGB ratio of 2.79, Figure 14a) and an average KAM value of 0.53° (Figure 14b). After prestraining the “A900” specimen, the fraction of LAGBs increased 1.5 times (to 42.1%). Accordingly, the HAGB:LAGB ratio decreased from 2.79 to 1.3, though HAGBs were still in the majority, leading to a lower KAM value compared to the “AsB” specimen (0.61° and 0.69°, respectively). In contrast to the “AsB” specimen, after post-prestraining annealing, the ratio of HAGBs and LAGBs in the “A900” specimen only changed slightly relative to prestraining: the HAGB:LAGB ratios were 1.69 and 1.33 for annealing at 900 °C and 1050 °C, respectively. As a result, in the “A900/P-S+1050” specimen, the KAM value was much higher than that of the “AsB/P-S+1050” specimen (0.50° and 0.30° respectively). These differences indicate that the thermally stabilised specimens (“A900s”) showed greater resistance of the worked-hardened structure to recrystallisation, which confirms the results above in the comparative study of the microstructure of the “AsB” and “A900” specimens (Figure 8 and Figure 10).

Figure 14c,d illustrate the EBSD results concerning the grain size evolution (the grain size was determined by an equivalent circle diameter). Following on from Figure 14, for all the processing modes, the grain size varied in a large range (from 2–6 µm to 100–110 µm); the particle size distribution had the form of a power law decreasing function, i.e., the highest fraction of the grains corresponded to its minimal diameter. In the “AsB” specimen, the fraction of the smallest grains was 20%; after prestraining and 900 °C annealing, it increased to 51.3% (due to the formation of new recrystallised grains), and it decreased to 42.9% under annealing at 1050 °C (due to the growth of recrystallised grains) (Figure 14c). Meanwhile, the average grain size gradually decreased from 20.6 µm (“AsB”) to 13.0 µm (“AsB/P-S+900”) and 10.0 µm (“AsB/P-S+1050”) (Figure 13d), reflecting the substitution of coarser work-hardened grains by smaller recrystallised ones. This complex evolution of a grain size was associated with the time-extended dynamics of the recrystallisation process, where the appearance of new grains was accompanied by the growth of previously formed recrystallised grains. In the specimens from the “A900” series, in contrast to the “AsB” specimens, the grain size distribution evolved insignificantly under prestraining and annealing: the highest fraction of the smallest grains varied within much narrower limits (32.4–38.7%), while the average grain size barely changed (17.0–18.8 µm). The noted grain size stability of the “A900” specimens complied with their more stable grain boundary state (LAGB/HAGB ratio, KAM) under different processing modes compared to the “AsB” specimens.

### 3.5. Structure of the Ultimately Deformed Zone and a Rupture Surface Characterisation

**Figure 15:**
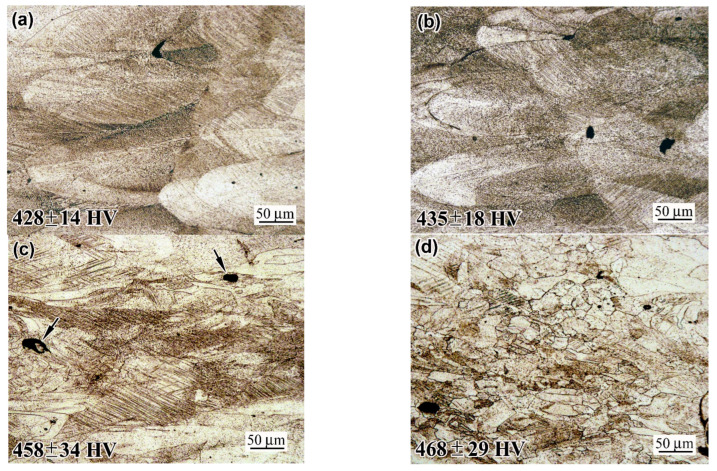
Microstructure and microhardness in the near-to-rapture zone of the tensile specimens: (**a**) “AsB”; (**b**) “AsB/P-S”; (**c**) “AsB/P-S+900”; (**d**) “AsB/P-S+1050”.

**Figure 16:**
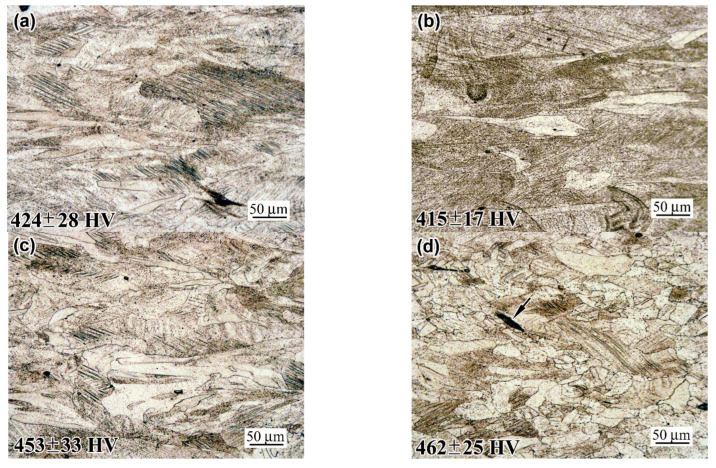
Microstructure and microhardness in the near-to-rapture zone of the tensile specimens: (**a**) “A900”; (**b**) “A900/P-S”; (**c**) “A900/P-S+900”; (**d**) “A900/P-S+1050”.

Figure 15 and Figure 16 show the ultimately strained microstructure of the neck of the fractured specimens at a distance of 0.5 mm from the rupture surface. The “AsB” and “AsB/P-S” specimens are seen to have similar structures, representing heavily stretched melt pools and bearing multiple slip bands located at an angle of approximately 45° to the strain direction. In some places, elongated ruptures of up to 50 µm in length can be seen (indicated by arrows in Figure 15c and Figure 16d), resulting from the strain-induced opening of the “lack-of-fusion” pores. In the structure of the “AsB/P-S+900” specimen, the “melt pool” contours are not revealed; the structure acquired a heavily deformed “laminate-type” pattern featuring multiple slip bands. The structure of the “AsB/P-S+1050” specimen contained small recrystallised grains deformed at an aspect ratio of 1:2–1:7 (notably, they contained fewer slip bands than the surrounding work-hardened matrix). The specimens “A900”, “A900/P-S”, and “A900/P-S+900” performed similar laminated patterns of heavily strained grains (Figure 15); no recrystallised grains were seen in the structure of the “A900/P-S+900” specimen. In contrast, the specimen “A900/P-S+1050” exhibited recrystallised grains deformed at an aspect ratio of 1:1.5–1:5 (Figure 16d).

The microstructure images presented in Figure 15 and Figure 16 are provided with corresponding microhardness values. These values (428–468 HV for the “AsB” group and 415–462 HV for the “A900” group) significantly exceeded the initial microhardness of the specimens by almost twofold (264 ± 9 HV of “AsB” and 222 ± 11 HV of “A900”), manifesting an intensive work-hardening phenomenon in the necking area of the specimens. It is noteworthy that the prestrained and annealed specimens were characterised by a higher microhardness relative to the specimens in the initial and prestrained states. This complies with the higher work-hardening capacity highlighted for the annealed specimens in Section 3.1 (Figure 4c).

**Figure 17:**
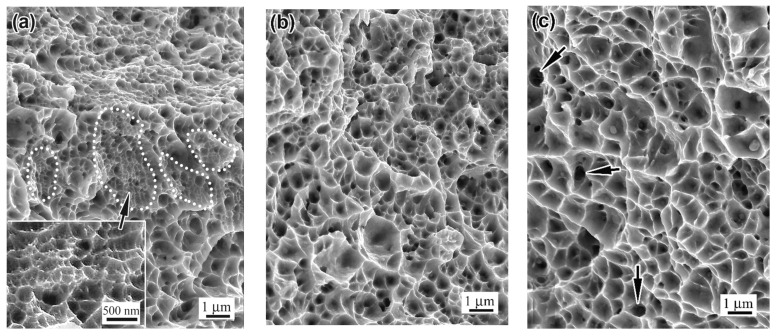

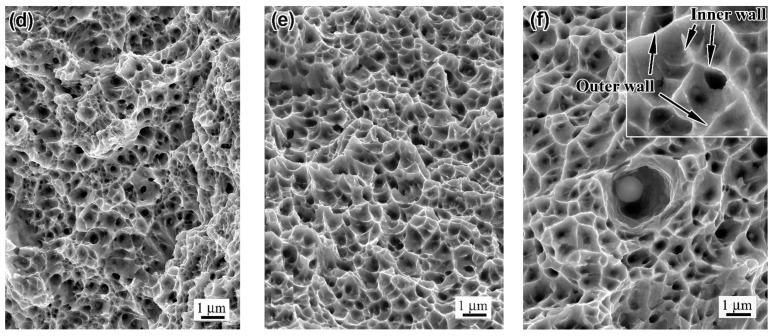
The rupture surface of the tensile specimens: (**a**) “AsB”, (**b**) “AsB/P-S”; (**c**) “AsB/P-S+1050”; (**d**) “A900”; (**e**) “A90/P-S”; (**f**) “A900/P-S+1050”.

The character of the tensile specimen rupture is illustrated by the images presented in Figure 17. All specimens in both groups, regardless of the processing mode, fractured via a ductile mechanism, with significant elongation under the necking (Figure 2). Elements of brittle failure, such as quasi-cleavage facets or a river pattern, were not observed in any specimen (Figure 17a–f). The fractured surface was composed of a large number of dimples, bordered by edge ridges formed due to the plastic flow of the metal. The dimples were characterised by their small size, varying in the range 0.05–1.50 µm; the majority of dimples had a diameter of less than 0.5 µm. Most pits had smooth bottoms but, in some of them, roundish pores were present that extended deeply into the specimens (shown by the arrow in Figure 17c), presumably as a result of the LPBF process.

The fracture structure featured a certain hierarchy. On the surface, relatively large dimples were found, bordered by high ridges (outer walls); inside them, the space was divided by lower ridges (inner walls), demarcating the shallower dimples with smaller diameters (insert in Figure 17f). Another characteristic feature of the fracture of the specimens (except the recrystallising-annealed ones) was the presence of areas of ultra-fine dimples (denoted in Figure 17a by the dashed line and shown in the insert in Figure 17a). The size of the dimples in these zones varied from 0.05 to 0.25 µm, with an average size of 0.14 µm, and about half of them were smaller than 0.2 µm (Figure 17d). Furthermore, the nucleation of the dimples on the spherical non-metallic inclusions was observed (Figure 17f).

## 4. Discussion

### 4.1. Evolution of Microstructure and Crystalline Features Under Applied Processing

The mechanical behaviour of the specimens can be determined based on the evolution of the crystal lattice’s state (the crystalline size, microstrain, and dislocation density). For this purpose, the FWHM values (Figure 11c) were analysed according to the Williamson–Hall method [58]:

(3) $$\beta_{hkl}\cos\theta =\frac{K\lambda }{D}+4\varepsilon \sin\theta$$

where *β_hkl_* is the FWHM, *D* is the size of coherent scattering domains, *K* is a shape factor (0.891), *λ* is the wavelength of Cu-K_α_ radiation, *θ* is the diffraction angle, and *ε* is the crystal microstrain. These are calculated as follows:

(4) $$\varepsilon =\frac{\beta_{hkl}}{4\tan\theta }$$

The average crystal size was found by Debye–Scherrer’s equation [59]:

(5) $$D=\frac{K\lambda }{\beta_{hkl}\cos\theta }$$

**Figure 18:**
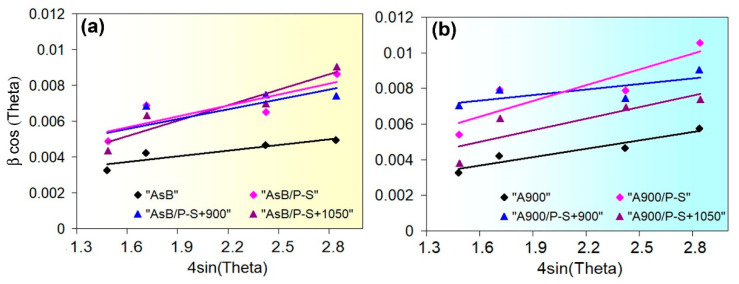
Williamson–Hall plots for the specimens of the (**a**) “AsB” group and (**b**) “A900” group.

The experimental values of *D* and *ε* were derived from the Williamson–Hall plots built into the axes of “4sin(*θ*)” (the X-axis) and “*β_hkl_*cos(*θ*)” (the Y-axis) (Figure 18). The crystal size was estimated from the Y-intercept of a linear fit to the experimental points, while the strain value was found as the slope of the fit to the X-axis.

The dislocation density was calculated by the Williamson–Smallman formula [60]:

(6) $$\rho_{XRD}=\frac{\sqrt{3k}\varepsilon }{Db}$$

where *ρ_XRD_* is the dislocation density detected by the X-ray diffraction method, *D* and *ε* are the parameters mentioned above, *b* is the magnitude of the Burgers vector of dislocations (*b* = 2.58 × 10^−10^ m [61]), and *k* is a coefficient, which is taken to be 1.2 [62].

The results of the calculations are presented in Table 2. It can be seen that the crystal lattice parameter (constant) hardly depends on the processing mode, varying only in the third decimal place. Regarding other structural features derived from XRD line broadening, both groups of specimens tend to vary. In the initial state, the “AsB” and “A900” specimen exhibited dislocation densities of 9.49 × 10^13^ m^−2^ and 7.91 × 10^13^, respectively, and almost the same microstrain (11 × 10^−4^–13 × 10^−4^). After prestraining, *ε* increased by 1.6–2.0 times, relative to the initial state, which corresponded to an increase in *ρ_XRD_* and a sharp decrease in *D*. The 900 °C annealing of the prestrained “AsB” and “A900” specimens resulted in a partial relief of microstrain, associated with a significant decrease in *ρ_XRD_*, while the crystal size increased by approximately 1.5 times. The annealing of the “AsB” specimen at 1050 °C resulted in an increase in microstrain and *ρ_XRD_*. In contrast, the same treatment of the “A900” specimen led to a further decrease in *ρ_XRD_* (increase in *D*), while the microstrain barely changed relative to 900 °C annealing. Notably, at any processing mode, the specimens of “AsB” group were distinguished by their smaller crystal size. 

The obtained data were compared with the results of the calculation of the geometrically necessary dislocation (GND) density. In contrast to the statistically stored dislocations (SSDs), GNDs result from deformation gradient fields as an accommodation of lattice curvature [58]. Thus, GNDs are used for the characterisation of the deformation process and strain hardening rate. The density of GNDs (*ρ_GND_*) was calculated based on the EBSD misorientation data [63,64,65] using the relationship proposed by Kubin and Mortensen [66]:

(7) $$\rho_{GND}=\frac{\alpha \theta }{\left|b\right|\Delta x}$$

where *θ* is the mean misorientation detected at the distance of ∆*x*, *b* is the magnitude of the Burgers vector, and *α* is a constant depending on the grain boundary type. For the *θ* and ∆*x*, the KAM mean value and a step size of EBSD measurements (0.03 µm) were used as a first-order approach. We also assumed *α* = 3, as proposed by Konijnenberg et al. [63], for the simple low-angle tilt grain boundary.

**Figure 19:**
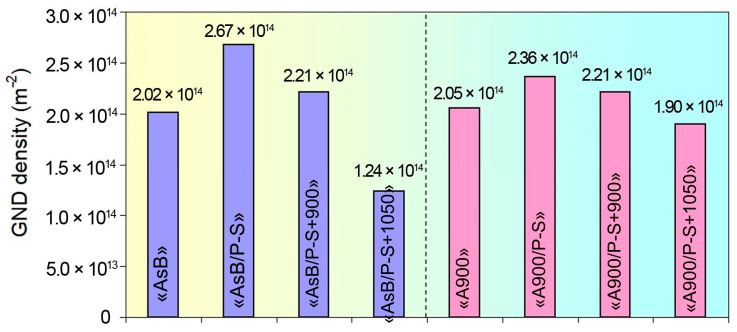
The evolution of a GNDs’ density depending on the processing mode.

As shown in Figure 19, the GND density varied in the range from (1.29–2.78) × 10^14^ m^−2^ to (1.98–2.46) × 10^14^ m^−2^ for the specimens of groups “AsB” and “A900”, respectively. These values are lower than those presented in Table 2 due to the fact that the XRD method detects the total dislocation density, encompassing both GHDs and SSDs. The highest values correspond to prestraining (resulting from maximum local grain misorientation), while the lowest *ρ_GND_* values were attributed to the annealing at 1050 °C. As observed, the *ρ_GND_* evolution generally coincided with the trend in ρ derived from the XRD study (Table 2), which is associated with the static recrystallisation process accompanied by the annihilation of lattice imperfections and the formation of non-deformed grains [43,67]. There is only one discrepancy regarding the 1050 °C annealing of the “AsB” specimen: the *ρ_GND_* reached a minimum value, while the *ρ_XRD_* value increased compared to annealing at 900 °C. This discrepancy is presumably connected with an elastic interaction of enlarged recrystallised grains and the untransformed matrix [68], resulting in heterogeneous deformation and strain localisation [69].

### 4.2. “Mechanical Properties-Microstructure” Correlations

The as-built specimens exhibited a unique cellular structure directionally crystallised under ultrafast melt pool cooling (Figure 7b,c). In LPBF Cr-Mo-containing alloys (Co-28Cr-6Mo, 316L), the cell boundaries are dislocation tangles associated with Cr and Mo segregations (both of which contribute to the strength of the LPBF alloys) [43,55,70]. During the LPBF, the “arched”-shape austenite grains form, following the “melt pool” shape (Figure 12a). During post-LPBF thermal stabilisation, the as-built cellular microstructure was disrupted, although the EBSD-revealed grain pattern and LAGB/HAGB ratio were retained (Figure 14a). Demolishing a cellular structure caused a noticeable drop in the YTS, indicating the significant role of the cell boundaries in hindering the dislocation motion. The prestraining process expectedly strengthened both the as-built and the thermally stabilised specimens, while their YTS increased to a greater extent. This was a result of specific changes in the crystalline structure—(i) an increase in the dislocation density (Table 2, Figure 18) and (ii) the appearance of microstrain—associated with the refinement of the coherent scattering domains. Microscopic observations revealed that the microstructure barely changed, featuring the slight elongation of the melt pools and the appearance of occasional dislocation slip bands (Figure 8a,c). Rare localised slip bands emerged in the most active slip systems, complying with the Schmid factor [71].

Subsequent 900 °C annealing of the prestrained “AsB” specimen led to the disappearance of the cellular/columnar structure (similar to the post-LPBF thermal post-stabilisation) and also resulted in the appearance of a low amount (7.4%) of small recrystallised grains, unevenly distributed in the work-hardened matrix (Figure 8d). Despite minimal structural changes, there was a significant XRD-detected reduction in microstrain and dislocation density (Table 2), which can be attributed to the simultaneous development of recovery and static recrystallisation processes. It should be noted that at this stage, in the “AsB” specimen, the number of LAGBs increased 1.4 times (Figure 14a), i.e., the formation of recrystallised grains began with the retention of coherence at the boundary with the work-hardened matrix. In contrast, the 900 °C annealing of the prestrained “A900” specimen did not visually alter the microstructure (Figure 10b), although it caused crystalline changes similar to those of the prestrained “AsB” specimen (Table 2). This allowed us to conclude that the alteration of mechanical properties of the “A900/P-S+900” specimen was primarily caused by the recovery process. Annealing at 1050 °C facilitated the more extensive development of recrystallisation in the “AsB” specimen, as evidenced by a 7-fold increase in the number of recrystallised grains (up to 49.7%) (Figure 9a) combined with an increase in their size (Figure 9b). As recrystallised grains grew, they lost coherence at the boundary with the matrix, as indicated by a sharp increase in the number of HAGBs (up to 82.3%). Consequently, heterogeneity in emerged strain distributions, which could cause slight increases in XRD-detected microstrain and dislocation density, as noted above (Table 2). In the prestrained specimen of the “A900” group, recrystallisation at 1050 °C encompassed only 19% of the volume, while the proportion of low-angle boundaries remained relatively high (43%), which is characteristic of the initial stage of recrystallisation. Ultimately, in the “A900/P-S+1050” specimen, the microstrain remained unchanged while dislocation density decreased by 1.5 times, compared to annealing at 900 °C.

It is of interest to compare the studied LPBF 316L steel with its analogue, produced by conventional metallurgical technology, with regard to the kinetics of static recrystallisation. Relevant data can be found elsewhere [72,73]. For instance, Kheiri et al. [72] applied recrystallisation annealing (at 850 °C, 950 °C, and 1050 °C) to commercial sheet cold-rolled 316L steel (with 70% reduction). They found that a fully recrystallised structure was achieved in steel after 1800 s, 90 s, and 60 s of holding, respectively (similar results were obtained by Du et al. [73]). In contrast, the present study demonstrated that after 1 h of holding at 1050 °C, the recrystallisation did not exceed 50%. Approximately the same result (55% at 1100 °C) was reported in [44] for the LPBF 316L steel in an as-built state. Consequently, in LPBF 316L steel, recrystallisation proceeds significantly more slowly than in conventional steel. The sluggish kinetics of recrystallisation in the AM-fabricated 316L steel has also been reported in [45,74]. This may be attributed to the higher degree of cold deformation applied in the aforementioned works, compared to the present study, which would result in a higher stored elastic energy. However, the primary reasons are probably the significant differences in the manufacturing technology and the initial structure of the steel. For example, Pinto et al. [74] explained the low recrystallisation rate of the LPBF 316L by the presence of multiple non-metallic nanoparticles, specifically rhodonite-like silicate (MnSiO_3_). In contrast, de Sonis et al. [44] considered the LAGBs the main factor controlling the recrystallisation kinetics in AM 316L steel. The reasons for the delayed recrystallisation in the LPBF steel require further clarification.

Furthermore, the presented data revealed the different behaviours of two studied groups of specimens regarding the recrystallisation rate. This behaviour depends on the processing history before prestraining and recrystallisation annealing. It was shown that the post-LPBF thermal stabilisation significantly inhibited static recrystallization in prestrained LPBF 316L steel, shifting its onset by approximately 150 °C relative to the as-built state. Accordingly, the specimens of an “A900” group exhibited higher values of YTS and indentation hardness with reduced TEL and AR at both annealing temperatures (Figure 4d,e). The increased susceptibility of the “AsB” specimens to recrystallisation can be explained by the as-built cellular structure, which ensures the more effective accumulation of dislocation under prestraining [74], as evidenced by the higher dislocation density revealed by the XRD and EBSD methods (Table 2, Figure 18). The stored elastic energy provided a greater driving force for the nucleation of recrystallised grains.

The obtained results should be considered in relation to improvements in LPBF 316L steel’s mechanical properties. As shown above, prestraining effectively hardened 316L steel but caused a 30–45% decrease in its ductility. Therefore, additional annealing is necessary to ensure the required evolution of the cold-worked structure to achieve a preferable strength/ductility balance. As evident from the data in Figure 4, prestraining and subsequent annealing at 900–1050 °C resulted in an increase in the ultimate tensile strength of 316L steel by 13–54 MPa relative to the as-printed state, with higher UTS values being characteristic of the “A900” group specimens (Figure 4b). At the same time, the total elongation decreased somewhat (to 49–53%) relative to the initial states while still remaining much higher than that of the ASTM F3184-15 requirements. After recrystallisation, the prestrained specimens acquired a much higher strain hardening capacity, as indicated by the (UTS–YTS) and YTS/UTS values (Figure 4c). To assess the strength/ductility combination of advanced high-strength steels (AHSS), the PSE parameter is widely used, which is the product of the ultimate strength and total elongation [75]. The PSE values for experimental samples of 316L steel are shown in Figure 4f. In the as-built state, LPBF 316L steel exhibited a PSE of 38.20 GPa·%. Among all the experimental specimens, the highest PSE value (44.48 GPa%) was observed in the post-LPBF thermally stabilised specimen, largely due to its exceptional TEL value (68%). Among the processing options that provide a higher yield strength, the specimens “AsB/P-S+1050” and “A900/P-S+900” stand out, with PSE values of 40.30 GP·a% and 38.37 GPa·%, respectively. Some of the PSE values (40.3 GPa·% and 44.48 GPa·%) comply with a target value (≥40 GPa·%) for the 3D generation of AHSSs [76] (referring to the feasibility of steel use in the production of high-strength components involving the deep drawing process [77,78]). Specimens of both groups, subjected to prestraining without subsequent annealing, were characterised by minimum PSE values (30–32 GPa·%), caused by their lower ductility (TEL of 39–42%).

### 4.3. Work-Hardening Behaviour and Fracture Mechanism

During the tensile test, the LPBF 316L steel specimens experienced significant work hardening, which is typical for alloys with an FCC structure [79]. As evident from Figure 4c, the specimens subjected to various processing modes differed notably in their strain-induced strength gain (UTS–YTS), with values ranging from 86 to 293 MPa. Thus, for most of the specimens (in the initial state, after annealing at 900 °C and 1050 °C), the work-hardening phenomenon significantly contributed to the ultimate tensile strength of steel. The hardening behaviour of a metal under plastic deformation can be analysed using strain hardening rate (*SHR*), which is derived from the engineering tensile curve [80]:

(8) $$SHR=\frac{d\sigma }{d\varepsilon }$$

where *σ* is the true stress and *ε* is the true strain.

The increase in stress *σ* with strain *ɛ* is considered to be a strain hardening phenomenon, while the hardening rate refers to the slope of the (*dσ*/*dɛ*) curve with respect to the strain axis. The *SHR* curves are presented in Figure 19 for both groups of specimens (only the part of the curves corresponding to plastic strain is shown).

**Figure 20:**
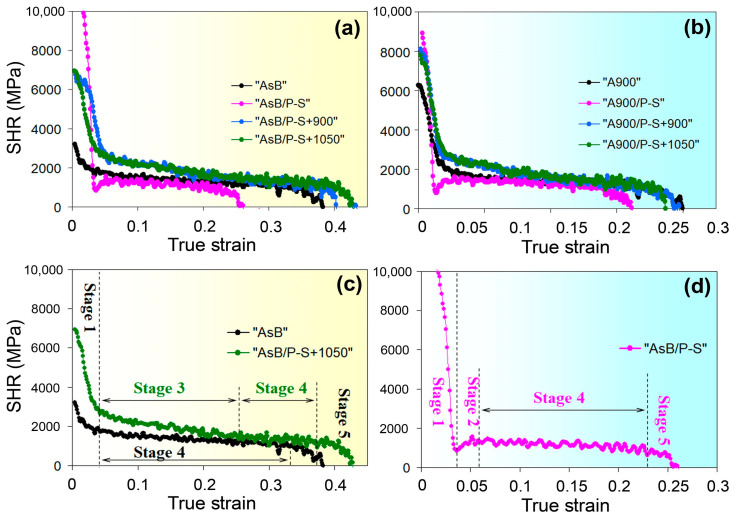
Work-hardening rate curves for the specimens of the groups (**a**) “AsB”b and (**b**) “A900”. The stages of the strain hardening of the specimens: (**c**) “AsB”; “AsB/P-S+1050”; and (**d**) “AsB/P-S”.

As observed in Figure 20, the presented *SHR* curves exhibit distinct patterns consisting of characteristic sections corresponding to specific stages of strain hardening. Under an elastic strain, *SHR* increased sharply; the maximum (11,150 MPa) and minimum (3200 MPa) *SHR* values were observed in specimens “AsB/P-S” and “AsB”, respectively (Figure 20a). Under a plastic strain, in the first stage of strain hardening, the *SHR* curves sharply declined with increasing strain to approximately 0.03–0.04 (Figure 20b) (this *dσ*/*dɛ* drop was caused by gliding dislocations and their cumulative interactions [80]). In the prestrained and annealed specimens, stage 1 was followed by the extended stage 3, at which the reduction in a strain hardening rate slowed down significantly (the angle of the curve’s slope decreased sharply) (Figure 20c). According to Zhang et al. [81], in 316L steel, the stage 3 in the *SHR* curve corresponds to the twin formation; thus, newly formed twins hindered the dislocation movement, thereby contributing to the work-hardening phenomenon. Stage 3 was followed by the near-plateau stage 4 (with a nearly constant *SHR* value), connected with a saturation of twins formation [81]. Subsequently, stage 4 transitioned to stage 5, at which *dσ*/*dɛ* decreased sharply to zero due to the slowdown of twinning activity [82] and dynamic recovery (the rearrangement of dislocation) [83]. The presented *SHR* profile is consistent with previously reported results [84] dedicated to the work-hardening mechanism in stainless steels (AISI 316, AISI 301).

In the *SHR* curves of the specimens in the initial state (“AsB”, “A900”), stage 3 was practically absent, presumably due to the obstruction of twinning in the fine cellular as-printed structure. Similar behaviour was observed by Karthik et al. [85] in LPBF-manufactured CuSn alloy; they concluded that the cellular structure significantly suppressed the twinning deformation by reducing homogeneous slip length and increasing stacking fault energy. The *SHR* curves of the prestrained specimens (“AsB/P-S” and “A900/P-S”) exhibited a different trajectory compared to those described above. In the first stage, *SHR* decreased to the greatest extent (down to 800–920 MPa) compared to other specimens. The prestrained specimens featured in stage 2, within a narrow strain range (∆*ε* = 0.02–0.03), led to a 500–700 MPa increase in *SHR*. Stage 2 was primarily attributed to the deformation-induced martensite transformation γ(FCC) → α′(BCC) [86], which might occur due to the increased dislocation density caused by prestraining (according to [87], the crystal imperfections decrease the activation energy of martensite nucleation due to local elastic stress accumulation]). Since the increase in *dσ*/*dɛ* at stage 2 was rather small, this indicated a rapid exhaustion of the places where α′-martensite could appear, i.e., the martensitic transformation made a limited contribution to the strength of prestrained specimens. In the “AsB” specimen, stage 2 was immediately followed by stage 4, which was also associated with the possible hindering of the twinning process by the cellular structure. The area under the *SHR* curve can be used to assess the energy consumed by the work-hardening process. Based on the shape of the *SHR* curves (Figure 20), this energy was higher for the specimens that underwent prestraining followed by recrystallisation annealing. This indirectly manifests their greater capacity for strain-induced hardening, which corresponds to the data presented in Section 3.1, as shown in Figure 4c.

The study of the near-fracture zone showed that the as-built microstructure retained its main morphological features (melt pool areas, cellular pattern) up to the ultimate deformation before rupture (Figure 15a,b). In the thermally stabilised or recrystallised specimens, the microstructure acquired a different pattern of a highly textured laminate, indicating ultimate strain hardening and the exhaustion of plasticity. Consequently, LPBF 316L steel gained higher strength and microhardness (the latter increased almost twofold compared with non-deformed areas). However, regardless of the processing mode, the fracturing of the tensile specimens occurred through a ductile mechanism (Figure 17), with void nucleation and coalescence leading to the appearance of isometric dimples with tear edges. The voids formed on non-metallic inclusions (as shown in Figure 17f), but in most cases they originated within the grain based on crystalline imperfections.

**Figure 21:**
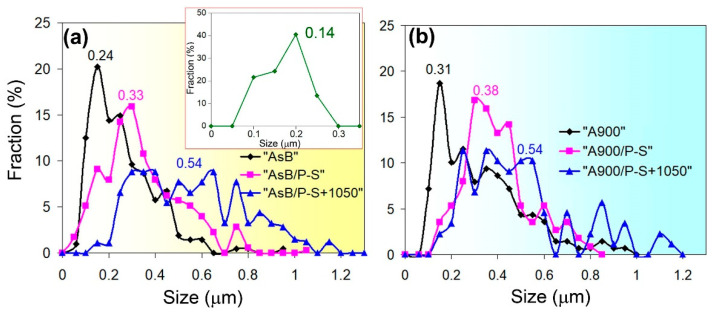
The distribution of the dimple size on the fractured surface in the specimens of groups: (**a**) “AsB” and (**b**) “A900”.

The ductile properties of the steel can be strongly attributed to the size, depth, and distribution of dimples on the rupture surface [61]. Figure 21 shows such a distribution for different groups of specimens and processing modes. As observed, the as-built specimen exhibited the smallest dimples (size range of 0.1–1.0 µm, with an average of 0.24 µm). Within its fractured surface, there were areas with ultra-fine dimples of 0.05–0.25 µm (in size) and an average value of 0.14 µm (also previously found by Guo et al. [56] in the fracture surface of 316L steel additively fabricated by electron beam freeform process). Since these values are similar to the cross-sectional size of columnar crystals, it suggests that the ultra-fine dimples originated within the columnar crystals (cells), preferentially oriented relative to the strain direction. A large number of cells predetermined a high density of the void nucleation sites, while the short *free* path of gliding dislocations inside the cell caused the rapid critical accumulation of dislocations necessary for micro-void nucleation. The simultaneous development of multiple micro-voids with a ductile breakup at the cell boundaries (resulting in edge ridges) hindered the preferential growth of some of them, thus preventing the formation of larger dimples. Post-LPBF thermal stabilisation and prestraining increased the average size of the dimples to 0.31–0.38 µm (Figure 21a,b). After prestraining and annealing, the average dimple size increased to 0.54 µm, while the upper limit of the values scatter extended to 1.2 µm. This shows that, as recrystallisation progressed and the density of crystalline imperfections decreased, the number of potential sites for the origin of voids decreased, and thus they could not grow to larger sizes.

Our study reports on the structure evolution in of LPBF 316L steel under prestraining and subsequent annealing, and emphasises the effect of the as-built LPBF structure on controlling the static recrystallisation process. It was demonstrated that recrystallisation products exhibited rather sluggish kinetics compared to wrought 316L steel and could even be slowed down by the post-LPBF thermal stabilisation, which transformed the structure from cellular to polyhedral. However, the obtained results have shown that prestraining and annealing can be utilised to tailor the mechanical property combination of the LPBF 316L steel with the aim of improving yield tensile strength and hardness by controlling the recrystallised grain fraction and dislocation density.

## 5. Conclusions

This study investigated the microstructural evolution and mechanical properties (under tensile testing and nanoindentation) of LPBF-manufactured 316L steel subjected to prestraining and subsequent annealing. The following key findings were observed:

1. As-printed microstructure: The as-printed 316L steel exhibited a hierarchical microstructure of melt pools with a cellular structure. This cellular structure consisted of the columnar crystal bundles averaging 0.2–1.5 µm in cross-sectional size. Post-LPBF thermal stabilisation at 900 °C eliminated the cellular structure, decreasing yield tensile strength (YTS) and hardness 1.4–1.5 times and increasing total elongation by 11 points compared to the as-built state.

2. Effect of prestraining: Prestraining with 0.12 plastic deformation significantly increased YTS by 25–65% and less noticeably increased ultimate tensile strength (UTS) by 15% while reducing steel ductility 1.5–1.6 times. The as-built steel’s main morphological features (melt pools, columnar crystals, cells) largely remained the same until tensile specimen rupture. Regardless of structure (processing mode), fracture occurred via a ductile mechanism, forming fine (0.1–1.2 µm) and ultra-fine (0.05–0.25 µm) isometric dimples. Dimple size in the as-built specimen correlated with the cross-sectional size of the columnar crystals.

3. Effect of annealing: One-hour annealing of prestrained LPBF 316L steel at 900 °C and 1050 °C initiated recovery and static recrystallisation. This decreased strength (more in YTS and less in UTS and indentation hardness) and increased ductility. The recrystallisation behaviour of work-hardened steel was influenced by its initial state (before prestraining). Post-LPBF thermal stabilisation hindered static recrystallisation and increased its onset temperature. In thermally stabilised specimens (“A900” group), recovery only occurred during 900 °C annealing, and low numbers of recrystallised grains (19%) were observed after 1050 °C annealing.

4. Mechanical property optimisation: Prestraining and subsequent annealing increased LPBF 316L steel’s UTS and hardness to 683–724 MPa and 3.9–4.2 GPa, respectively, maintaining total elongation above 50%. In the “A900/P-S+1050” specimen, the product of strength and elongation (PSE) (40.3 GPa·%) exceeded that of the as-built state. Maximum PSE value (44.5 GPa·%) was observed in specimens that were thermally stabilised after LPBF.

5. Strain hardening behaviour: Prestraining and subsequent annealing enhanced the strain hardening capacity of LPBF 316L steel. In the prestrained specimens (“AsB/P-S” and “A900/P-S”), a deformation-induced martensitic transformation may occur at the early stage of plastic deformation (*ε* = 0.02–0.03), contributing to strength increases.

## Figures and Tables

**Table 1:** Chemical composition (wt.%) of the LPBF 316L steel studied in this work.

C	Si	Mn	P	S	Cr	Ni	Mo	Cu	V	Ti	Nb	Co	Al	Fe
0.022	0.80	1.08	0.018	0.007	16.39	11.92	2.36	0.15	0.05	0.006	0.04	0.10	0.02	bal.

**Table 2:** The lattice parameter and quantitative indicators of line broadening.

Specimens	Lattice Parameter (Å)	Microstrain	Crystalline Size (Å)	Dislocation Density (m^−2^)
The specimens of an “AsB” group				
«AsB»	3.597	11.1 × 10^−4^	860.0	9.49 × 10^13^
«AsB/P-S»	3.597	20.0 × 10^−4^	345.2	4.26 × 10^14^
«AsB/P-S+900»	3.596	5.1 × 10^−4^	557.7	6.73 × 10^13^
«AsB/P-S+1050»	3.598	22.0 × 10^−4^	966.7	1.67 × 10^14^
The specimens of an “A900” group				
«A900»	3.598	13.0 × 10^−4^	1208.2	7.91 × 10^13^
«A900/P-S»	3.599	21.3 × 10^−4^	433.3	3.62 × 10^14^
«A900/P-S+900»	3.599	8.2 × 10^−4^	651.4	9.26 × 10^13^
«A900/P-S+1050»	3.599	9.0 × 10^−4^	1102.3	6.00 × 10^13^

## Data Availability

The original contributions presented in this study are included in the article. Further inquiries can be directed to the corresponding author.
